# Association Between Serum Antinuclear Antibody and Polycystic Ovarian Syndrome in Reproductive Women Aged 18 to 35 Years: A Quest for an Autoimmune Marker

**DOI:** 10.7759/cureus.75224

**Published:** 2024-12-06

**Authors:** Chandrashekhar Shrivastava, Praharshitha Sagiraju, Sarita Rajbhar, Ruchi Bansal, Loukya Kodumuri

**Affiliations:** 1 Obstetrics and Gynaecology, All India Institute of Medical Sciences, Raipur, IND

**Keywords:** antinuclear antibody, auto immune, modified ferriman-gallwey, poly cystic ovarian disease, rotterdam criteria

## Abstract

Introduction: Polycystic ovarian syndrome (PCOS) is a heterogeneous endocrinal physiological disorder characterized by chronic oligo-ovulation or an-ovulation, hyperandrogenism, and polycystic morphology in ovaries on transvaginal or abdominal ultrasound. Hyperandrogenism and insulin resistance are already well-documented pathophysiological mechanisms in PCOS. Besides this, autoimmunity has been hypothesized in its pathogenesis. Studies regarding the association between PCOS and autoimmunity have yielded contradictory results. There is scarce data regarding the prevalence of autoimmune markers among PCOS women from India. Therefore, this study was done to find the relationship between antinuclear antibody (ANA) positivity and PCOS.

Objective: To determine the prevalence of ANA positivity in women with PCOS and to see the association of Serum antinuclear antibodies ANA with clinical, hormonal, and biochemical parameters of PCOS.

Methods: This cross-sectional study was done in the Obstetrics and Gynaecology department, All India Institute of Medical Sciences (AIIMS), Raipur, for a period of 18 months. The study population consisted of a total of 140 women aged 18-35 years and were equally divided into the ‘PCOS ‘and ‘Control’ groups. The ‘PCOS’ group consisted of 70 women with a diagnosis of PCOS as per revised 2003 Rotterdam criteria. The ‘Control’ group consisted of 70 healthy women with no PCOS. History-taking and clinical examinations were done on all women. History taking included medical, surgical, menstrual, and any other relevant history. Clinical examinations included general physical examination and anthropometry like height, weight, build, body mass index (BMI), waist-to-hip ratio (WHI), etc. We did Hirsutism scoring with a modified Ferriman-Gallaway scoring system. Blood samples were taken for ANA estimation and biochemical and hormonal essay.

Results: The prevalence of ANA positivity in PCOS and the control group was 10% and 4.3%, respectively (not statistically significant). There was no significant association found between ANA positivity and PCOS (p-value=-0.326). There was no significant association found between ANA positivity and modified Ferriman-Gallwey scores, serum testosterone levels and HOMA-IR (Homeostatic Model Assessment for Insulin Resistance) levels.

Conclusion: There was no statistically significant association between ANA positivity and PCOS, but we found an increased prevalence of ANA in the PCOS group as compared to the non-PCOS group.Though our study did not find a significant association between ANA positivity and modified Ferriman Gallwey score, serum testosterone levels, and HOMA-IR levels, we found a significant increase in anti-TPO levels in ANA-positive PCOS women. We conclude that the increased prevalence of ANA positivity in PCOS could be an indicator of autoimmunity.

## Introduction

Polycystic ovarian syndrome is a heterogeneous endocrinal physiological disorder characterized by chronic oligo-ovulation or an-ovulation, hyperandrogenism, and polycystic morphology in ovaries on transvaginal or abdominal ultrasound. The prevalence of PCOS in India is 3.7 to 22.5% [1,2]. Worldwide, there is a substantial increase in the prevalence of PCOS nearly paralleling type 2 diabetes mellitus [3]. Hyperandrogenism and insulin resistance are already well-documented pathophysiological mechanisms in PCOS [4-6]. Besides this, autoimmunity has been hypothesized in the pathogenesis of PCOS [7]. Various studies have shown an increased incidence of systemic autoimmune diseases in women of reproductive age and hypogonadal men with relatively excess estrogens [8,9].

Although hyperandrogenism in PCOS patients appears to protect against the emergence of autoimmune diseases, various mechanisms associated with estrogenic reactions on the immune system contradict this. Progesterone could inhibit these estrogenic properties on the immune system, but because of oligo-ovulation or anovulation, there are low levels of serum progesterone leading to immune system overstimulation and autoantibody production [10].

The cause of PCOS is unknown exactly. Many environmental factors appear to interact intricately with genetic factors. Studies done regarding the association between PCOS and autoimmunity have yielded contradictory results [11, 12]. It is suspected that there may be autoantibodies that may affect their management [13,14]. As a result, the varying levels of autoantibodies in different PCOS patients provide a new avenue for future molecular research that may aid in the identification of better PCOS treatment options in the future.

There is scarce data regarding the autoimmune markers prevalence among PCOS women from India. So, the present study was carried out to determine the association between antinuclear antibody (ANA) positivity and PCOS.

## Materials and methods

This cross-sectional study was done in the Obstetrics and Gynaecology department, All India Institute of Medical Sciences (AIIMS), Raipur, for a period of 18 months after clearance from the Institute's Ethical Committee. The study population consisted of a total of 140 women aged 18-35 years and were equally divided into the ‘PCOS’ and ‘Control groups after applying inclusion and exclusion criteria. The ‘PCOS’ group consisted of 70 women with a diagnosis of PCOS as per the revised 2003 Rotterdam criteria. The control exclusion criteria group consisted of 70 healthy women with no PCOS. History taking and clinical examination were done in all women. We followed the World Health Organization's (WHO) STEPwise approach (WHO STEPS) protocol for behavioral risk factors, physical examination, and biochemical tests. History taking included medical, surgical, menstrual, and any other relevant history. Clinical examinations included general physical examination and anthropometry as per WHO, like height, weight, build, body mass index (BMI), waist-to-hip ratio (WHR), and waist and hip circumferences.

**Figure 1:**
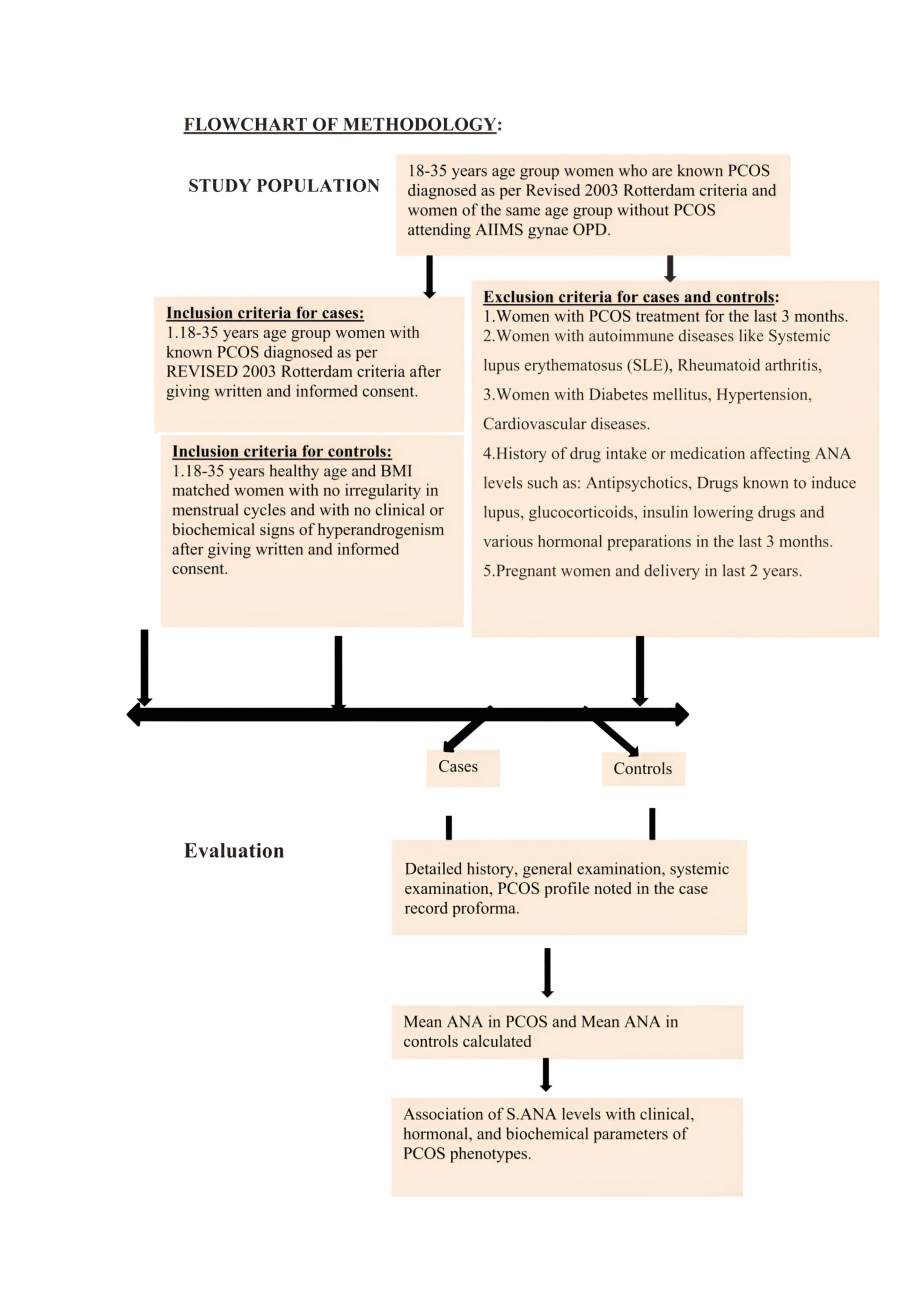
Flow chart of methodology.

A head-to-toe examination was done, including an examination of acanthosis nigricans, acne, alopecia, and facial and body hair. Type and grade of alopecia noted according to the Ludwig grading system. We considered androgenic pattern alopecia, a Ferriman-Gallaway score of >8 (hirsutism), and acne as clinical signs of hyperandrogenism. Elevated serum testosterone levels (>59 ng/ml) were considered as biochemical hyperandrogenism. Systemic examination, including respiratory, cardiovascular, central nervous system, per abdomen examination, thyroid, and breast examination, was done. USG for antral follicular count and ovarian volume were performed. Lab tests such as blood glucose (fasting and 2 hours postprandial), fasting insulin, serum LH (luteinizing hormone), FSH (follicle-stimulating hormone), thyroid-stimulating hormone (TSH), anti-TPO (thyroid peroxidase) levels, free testosterone, DHEAS (dehydroepiandrosterone sulfate), lipid profile, prolactin levels, and HOMA-IR (homeostatic model assessment for insulin resistance) were done. A fasting blood sample for antinuclear antibodies (ANA) estimation was taken. The prevalence of ANA in PCOS and controls was calculated. The association of serum ANA positivity was done with clinical, hormonal, and biochemical parameters of different PCOS phenotypes (Figure 1).

Inclusion criteria

For the PCOS Group

Women aged 18-35 years who had given written and informed consent for the study and who were already diagnosed with PCOS as per the revised 2003 Rotterdam criteria were included.

For the Control Group

Age and BMI-matched women with no irregularity in menstrual cycles and with no clinical hyperandrogenism or biochemical signs of hyperandrogenism were included.

Exclusion criteria

For Both the PCOS and Control Groups

Women with a diagnosis of androgen-secreting tumors, Cushing’s syndrome, congenital adrenal hyperplasia, hypothyroid, hyperthyroid disorders, and hyperprolactinemia. Women who were being treated for PCOS for the last 3 months, women with systemic lupus erythematosus (SLE), Sjogren’s syndrome, rheumatoid arthritis, and other autoimmune diseases, women with medical disorders like diabetes, hypertension, and cardiovascular diseases, women who had a history of taking antipsychotics (chlorpromazine, clozapine, haloperidol), which affects ANA levels, women who had a history of taking lupus-inducing drugs like pyrazinamide, sulfadiazine, and aromatase inhibitors, women who took corticosteroids, insulin-lowering drugs, and various hormonal preparations in the preceding 3 months, pregnant women and who had delivery in the last 2 years were excluded, as shown in Table 1. 

**Table 1:** Exclusion criteria and number of patients excluded as per different criteria.

Exclusion criteria	Number of patients excluded as per criteria
Women with PCOS treatment for the last 3 months	73
Women with autoimmune diseases like systemic lupus erythematosus, rheumatoid arthritis, sjogren syndrome	3
Women with diabetes mellitus	3
Women with hypertension	0
History of drug intake or medication such as antipsychotics	0
Glucocorticoids	0
Insulin lowering drugs(metformin)	2
Various hormonal preparations (other than OC pills) in the last three months	5
Pregnant women	0
Delivery in the last two years	2
Total number of patients excluded	88

Sample size calculation

Sample size was calculated using web-based Epitools Sample Size Calculator software based on data from a previously published study by Rashid et al. [15]. ANA positivity in the PCOS group was found to be 18.4% compared to 2.29% in the control group.

\[ p = \frac{p_1 + p_2}{2} = 0.103 \]

Where p_1_ is 0.184, p_2_ is 0.0229

\[ Z_{\alpha/2} = 1.96 \]

(at a 95% confidence interval)

\[ Z_{\beta} = 0.84 \]

(at 80% power)

After substituting the values into the formula, the total estimated sample size for both groups was 134. To simplify, this was rounded up to 140, requiring 70 participants per group.

Statistical analysis

We used IBM Statistical Package software for the Social Sciences (SPSS) version 22 for Windows. Continuous variables were expressed as mean±SD (standard deviation). Categorical variables were expressed as frequency and percentages. Two-sided p-values were considered statistically significant at p<0.05. Descriptive statistics calculations were done for age distribution, occupation, education, socioeconomic status, marital status, and menstrual cycles per year. Frequency and percentage were calculated for the clinical presentation of PCOS cases and FG score calculations. An independent t-test was done to compare anthropometric, clinical, biochemical, and hormonal characteristics between the PCOS group and controls. Again, an independent t-test was used to compare AFC and ovarian volume between PCOS and controls. A chi-square test was done to see the association of ANA positivity with serum testosterone level, HOMA-IR values, and modified Ferriman Gallwey score.

## Results

The mean±SD ages of the PCOS and controls were 24.90±4.24 years and 25.75±4.75 years, respectively, and were comparable. The mean WHR of PCOS patients was 0.98, and the controls were 0.93, which was statistically significant (p=0.002). We found a significant difference between the two groups regarding education, occupation, socioeconomic status, area of residence, and marital status with a p-value of <0.05. Infrequent menstrual cycles were the most common clinical presentation and were present in 56 (80%) women of the PCOS group. There was a significant difference between the mean FG score between PCOS (4.24) and control groups (0.10), with a p-value of <0.001. There was a significant difference in mean fasting blood sugar levels, serum testosterone levels, LH: FSH ratio, LH levels, and serum vitamin D levels between PCOS cases and the control group (p < 0.001). No significant difference was found in postprandial blood sugar levels, lipid profile, or 17 hydroxy progesterone levels between PCOS and control groups. In between the ANA-negative PCOS and ANA-positive PCOS groups, a significant difference (p-value of 0.05) was found in anti-TPO levels, while no significant difference was found for fasting insulin levels and HOMA-IR values. The prevalence of ANA positivity in PCOS was found to be 10%, which is higher than the prevalence of ANA positivity in healthy controls, which was 4.3%. We found no significant association between ANA positivity and PCOS (p=0.326). There was a significant association between anti-TPO levels and ANA positivity (p=0.05). In our study, there was no significant association between ANA positivity and modified Ferriman Gallwey scores, serum testosterone levels, and HOMA-IR levels (Tables 2-5).

**Table 2:** Comparative description of anthropometric, clinical, biochemical, and hormonal characteristics of pcos women vs controls. Independent t-test; *p<0.05 considered as significant; **p<0.001, considered as highly significant. BMI: body mass index, WHR: waist-hip ratio, LDL: low-density lipoprotein, HDL: high-density lipoprotein, TSH: thyroid stimulating hormone, DHEAS: dehydroepiandrosterone sulfate, FSH: follicle stimulating hormone, LH: luteinizing hormone, 17OH progesterone=17-hydroxyprogesterone.

Parameters	Variables	PCOS group N=70		Control group N=70		p-value (Unpaired t test)
		Mean	SD	Mean	SD	
Anthropometric	Weight (Kg)	57.77	11.08	59.21	10.21	0.425
	BMI (Kg/m2)	23.74	4.78	22.73	3.88	0.172
	WHR	0.98	0.12	0.93	0.02	0.002*
Clinical	Modified Ferriman Gallwey score	4.24	4.93	0.10	0.34	<0.001**
Biochemical	Fasting blood sugar (mg/dl)	97.14	8.98	90.87	9.00	<0.001**
	PP blood sugar (mg/dl)	116.91	27.62	122.24	13.22	0.149
	Cholesterol (mg/dl)	165.44	31.26	161.45	24.70	0.404
	LDL (mg/dl)	104.37	25.59	102.82	20.48	0.694
	HDL (mg/dl)	41.51	9.83	42.31	9.71	0.629
	Triglyceride (mg/dl)	103.72	52.40	94.31	32.22	0.203
Hormonal	TSH (µIu/ml)	2.57	1.53	2.26	0.74	0.131
	Prolactin (ng/ml)	9.93	4.96	9.00	5.85	0.313
	DHEAS (µg/dl)	215.35	125.07	191.51	101.03	0.217
	FSH (miu/ml)	7.58	5.59	6.92	1.18	0.606
	LH (miu/ml)	8.12	6.85	4.72	3.16	<0.001**
	LH: FSH (miu/ml)	1.40	0.90	0.57	0.07	<0.001**
	17-OH progesterone (miu/ml)	0.90	0.97	1.37	3.31	0.258
	Testosterone (ng/dl)	32.06	19.27	14.80	4.09	<0.001**
	Vit D (ng/dl)	15.17	6.47	37.81	12.87	<0.001**

**Table 3:** ANA positivity in PCOS women and controls.

ANA	PCOS women=70	Controls=70	Unpaired t-test
	N (%)	N (%)	
Positive	7 (10%)	3 (4.3%)	p-value: 0.326
Negative	63 (90%)	67 (95.7%)	

**Table 4:** Comparison of anti-TPO, fasting insulin, HOMA-IR in ANA-positive and ANA-negative PCOS cases. Anti-TPO: anti thyroid peroxidase, HOMA-IR: homeostatic model assessment for insulin resistance.

Variables	ANA-negative PCOS group	ANA-positive PCOS group	Total	p-value (unpaired t-test)
	Mean±SD	Mean±SD	Mean±SD	
Anti TPO	75.52±224.75	221.04±475.84	90.08±258.87	0.05
Fasting Insulin	11.05±6.49	14.97±11.27	11.44±7.10	0.399
HOMA-IR	2.63±1.57	3.30±2.50	2.69±1.68	0.513

**Table 5:** Comparison of mFG score, serum testosterone, HOMA-IR in ANA-positive and ANA-negative PCOS cases. mFG score: modified Ferriman Gallwey score, HOMA-IR: homeostatic model assessment for insulin resistance.

Variables	ANA-negative PCOS group	ANA-positive PCOS group	Total	p-value (chi-square test)
	N(%)	N(%)	N(%)	
mFG score (≥8)	18 (28.6%)	4 (57.1%)	22 (31.4%)	0.26
Serum testosterone (≥59)	6 (9.5%)	0 (0%)	6 (8.6%)	0.393
HOMA-IR (>2.5)	36 (57.1%)	6 (85.7%)	42 (60.0%)	0.14

## Discussion

Out of a total of 140 women enrolled in the study, 70 women had PCOS, and 70 were healthy controls. We found a 10% prevalence of ANA positivity in PCOS. A prevalence of 1.081%(4 out of 370 healthy blood donors ) was found for ANA positivity by Sharma et al [16]. Guo et al. found the prevalence of ANA in the general population to be around 5.92% in a very large sample size from China [17]. In our study, 7 out of 70 cases (10%) tested positive for ANA in the PCOS group, and 3 out of 70 subjects (4.3%) tested positive for ANA in the control group with a p-value of 0.326, suggesting no statistical difference between the two groups. A study by Rashid et al. found a statistically significant difference in the prevalence of ANA positivity in PCOS women (18.4%) vs the prevalence of ANA positivity among controls (2.29%) with a p-value <0.01 [15]. In a study by Hefler-Frischmuth et al., serum levels of ANA (optical density quotient) were similar in both the PCOS group and the Control group. In the same study, they found statistically significant elevations of anti-histone (p-value=0.02) and anti-dsDNA antibodies (p-value=0.02) (Table 6)[13].

**Table 6:** Prevalence of ANA in PCOS women in various studies.

Studies	Type of study	Sample size	Prevalence of ANA in PCOS cases
Present study	Cross-sectional	140	10%
Mobeen et al (2016)[12]	Systematic review	-	8.60%
Rashid et al (2018)[15]	Cross-sectional	167	18.40%

Increased prevalence of ANA in PCOS patients in our study as compared to healthy controls could possibly suggest autoimmunity as a possible etiology and further larger cohort studies are needed to know the exact pathophysiology behind autoimmune etiology in PCOS. There was a significant difference between the mean mFG score in PCOS (4.24) and control groups (0.10) with a p-value <0.001. ANA positivity and modified Ferriman Gallwey score were not significantly associated (p-value=-0.26) in our study.

Hyperandrogenism is known to have a dominant role in the pathogenesis of PCOS, in addition to insulin resistance. There is a need to evaluate the association of hyperandrogenism with autoimmunity, though our study did not show a significant association. Contrary to our study, Rashid et al. [15] found a significant correlation between ANA positivity and mean FG scores.

The mean level of anti-TPO in the ANA negative PCOS group was 75.52 with a standard deviation (SD) of 224.75, and in the ANA positive PCOS group was 221.04 with a standard deviation (SD) of 475.84 with a p-value of 0.05, which was statistically significant. This statistical significance could possibly signify the association of autoimmunity with PCOS. In a study by Arora et al., it was found that there was a higher prevalence of antithyroglobulin antibodies in PCOS women, suggesting that there could be a role of autoimmunity in PCOS pathogenesis [18].

Limitations

The primary limitation of the study is its relatively small sample size, which may impact the statistical power and generalizability of the findings. The design of our study is cross-sectional, so the possibility of establishing causality or temporal relationships between ANA positivity and PCOS is less. The study is mainly confined to the population of the small catchment area of the Raipur district of Chattisgarh state, so further research with a larger multicenter trial is needed to confirm findings in other Indian regions. 

## Conclusions

Even though it was not statistically significant, we found an increased prevalence of ANA positivity in PCOS women than in controls. Though our study did not find a significant association between ANA positivity and modified Ferriman Gallwey score, serum testosterone levels, or HOMA-IR levels, we found a significant increase in anti-TPO levels in ANA-positive PCOS women. So, tests of other markers of autoimmunity could open new areas of interest for understanding PCOS and its efficient management. It might be concluded that the increased prevalence of ANA positivity in PCOS could be an indicator of autoimmunity. It is not known whether PCOS women with ANA positivity are at greater risk of developing autoimmune diseases such as SLE, rheumatoid arthritis, etc., which should be investigated in further follow-up studies. 
